# RP-HPLC-ESI-QTOF-MS Qualitative Profiling, Antioxidant, Anti-Enzymatic, Anti-Inflammatory, and Non-Cytotoxic Properties of *Ephedra alata* Monjauzeana

**DOI:** 10.3390/foods11020145

**Published:** 2022-01-06

**Authors:** Latifa Khattabi, Tarek Boudiar, Mustapha Mounir Bouhenna, Aziez Chettoum, Farid Chebrouk, Henni Chader, Jesús Lozano-Sánchez, Antonio Segura-Carretero, Gema Nieto, Salah Akkal

**Affiliations:** 1 Faculty of Nature and Life Sciences, University of Brothers Mentouri, Constantine 1, BP, 325 Route de Ain El Bey, Constantine 25017, Algeria; azizchettoum@yahoo.fr; 2 National Center of Biotechnology Research Constantine (CRBt), Ali Mendjli Nouvelle Ville UV 03 BP E73, Constantine 25016, Algeria; boudiar_tarek@yahoo.fr; 3 Centre de Recherche Scientifique et Technique en Analyses Physico-Chimiques (CRAPC), BP384, Bou-Ismail, Tipaza 42004, Algeria; bouhenna3@yahoo.fr (M.M.B.); chebrouk.farid@crapc.dz (F.C.); 4 Departement of Pharmacy, Faculty of Medicine, University of Algiers 1, Algiers 16001, Algeria; henni.chader@univ-alg1.dz; 5 Research and Development of Functional Food Center (CIDAF), Bioregion Building, PTS Granada, Avda. De l Conocimiento s/n, 18016 Granada, Spain; jesusls@ugr.es (J.L.-S.); ansegura@ugr.es (A.S.-C.); 6 Department of Food Technology, Food Science and Nutrition, Faculty of Veterinary Sciences, Regional Campus of International Excellence “Campus Mare Nostrum”, Espinardo, 30071 Murcia, Spain; 7 Valorization of Natural Resources, Bioactive Molecules and Biological Analysis Unit, Department of Chemistry, University of Mentouri Constantine 1, Constantine 25000, Algeria; salah4dz@yahoo.fr

**Keywords:** *Ephedra alata* monjauzeana, chemical characterization, biological virtues, crude extract

## Abstract

An investigation was conducted to study the beneficial effects of *Ephedra alata* monjauzeana crude extract (EamCE). The chemical profile was determined using RP-HPLC–ESI-QTOF-MS analysis, revealing the presence of twenty-one flavonoids and phenolic acids. A series of antioxidant assays was carried out using ten different methods. The EamCE has demonstrated a significant antioxidant potential, with interesting IC_50_ values not exceeding 40 µg/mL in almost activities. Likewise, a significant inhibition of key enzymes, involved in some health issues, such as Alzheimer’s disease, diabetes, hyperpigmentation, dermatological disorders, gastric/urinary bacterial infections, and obesity, was observed for the first time. The IC_50_ values ranged from 22.46 to 54.93. The anti-inflammatory and non-cytotoxic activities were assessed by heat-induced hemolysis and cell culture methods, respectively; the EamCE has shown a prominent effect in both tests, notably for the anti-inflammatory effect that was superior to the reference compound “diclofenac” (IC_50_: 71.03 ± 1.38 > 70.23 ± 0.99 (µg/mL)). According to these results, this plant could be used in a large spectrum as a food supplement, as a natural remedy for various physiological disorders and pathologies; and it might serve as a preventive and health care agent.

## 1. Introduction

Plants have been exploited since archaic ages in all countries and cultures, for their potential medicinal properties. The great civilizations of the ancient Chinese, Indians, and North Africans left written evidence of man’s experience in employing plants for the cure of a full range of sicknesses [1,2,3]. In our study, we have opted for *Ephedra alata* monjauzeana, a plant used by the Saharawi people of Algeria’s desert as herbal tea to help them sleep, relax, calm their anxiety, and reduce stress, hence the interest in deepening the knowledge of this plant and support of its traditional therapeutic use. The foliage of *Ephedra alata* (*E. alata*) has a pleasant aroma and has been used as foodstuff for animal grazing in Saudi Arabia [4]; likewise, it presents a promising natural source of constituents that could be used as food additives [5,6]. Today, medicinal food plants are being prescribed in the form of complementary and alternative medicine therapies [7]. Indeed, natural products from plant origin, either as pure compounds or as standardized extracts afford durable source to design novel drugs [8,9,10]. *Ephedra* (family Ephedraceae, the joint firs) is a genus of non-flowering seed plants related to the Gnetales, the closest living relatives of the Angiosperms. Approximately 50 *Ephedra* species worldwide are shrubs adapted to semiarid and desert conditions. Around 25 species of *Ephedra* are encountered in the arid zones of the Old World spanning westwards from Central Asia across southwest Asia and into Mediterranean Europe and North Africa [11]. Moreover, *E. alata*, is a medicinal plant that grows mostly in the desert and is known to comprise approximately 40 species that populate dry environments; it has been frequently employed in traditional medicine in China and most Arabian countries, for diverse medical intents. In other respects, numerous secondary metabolites of *E. alata,* counting alkaloids, tannins, saponins, proanthocyanidins, phenolic acids, flavonoids, and essential oils, have been recorded, and the plants-derived polyphenols are of extreme interest for their high antioxidant activities [12,13]. In China, people used to apply it as a traditional medicine for 5000 years to treat allergies, bronchial asthma, chills, colds, coughs, edema, fever, flu, headaches, and nasal congestion [14].

The intentions of this first study on *E. alata* monjauzeana, taking into account earlier findings concerning other *E. alata* subspecies, were to confirm the same proven biological effects of *E. alata*. To the greatest extent, checking and characterizing new virtues and molecules that particularly define the plant of interest. 

## 2. Materials and Methods

### 2.1. The Chemicals

Standards compounds (purity ≥ 95%), chlorogenic acid, quercetin-3-O-glucoside, kaempferol-3-O-glucoside, kaempferide, luteolin-7-O-glucoside, and apigenin were purchased from Sigma Aldrich (St. Louis, MO, USA). Syringic acid was sourced from FlukaChemika (Buchs, Switzerland). Stock solutions were prepared at 1 mg/mL in methanol and properly diluted before analysis. Solvents used for extraction and analysis were of analytical and HPLC-MS grades, respectively. Methanol, acetonitrile, and formic acid were obtained from Fisher Chemicals (Thermo Fisher, Waltham, MA, USA). Ultrapure water was obtained by a Milli-Q system (Millipore, Bedford, MA, USA). Acetylcholinesterase (AChE) type VI-S, from electric eel b1000 U/mg solid, Butyrylcholinesterase (BChE) from equine serum 100 U/mg solid, 5,5′-dithiobis[2-nitrobenzoic acid] (DTNB), butyrylthiocholine chloride, galantamine, 2,2-diphenyl-1-picrylhydrazyl (DPPH), 2,2′azinobis-3-ethylbenzothiazoline-6-sulfonic acid (ABTS), linoleic acid, β-carotene, 2,6-di-tert-butyl-4-hydroxytoluene (BHA), tween 40, neocuproine, alpha amylase from aspergillus oryzae, 3-(3,4-dihydroxyphenyl-2,5,6-d3)-L-alanine (L-DOPA), kojic acid, thiourea, lipase from porcine pancreas, tyrosinase from mushroom ≥ 1000 U/mg solid, urease from Canavalia ensiformis (Jack bean) and other chemicals were Sigma Aldrich products and acetylthiocholine iodide was purchased from BioChemica.

### 2.2. Plant Material

The aerial parts of *E. alata* monjauzeana were collected during the flowering season from southeast of Algeria “Djebal Antar, Beni Abbes-Bechar”. The recovered quantity was stored in the departmental Herbarium (dark room at cool temperature) of the Biotechnology Research Center’s Health division. The plant was identified and authenticated according to the phenotypical features described by Dubuis and Faurel, in 1957 [15].

### 2.3. Extraction

The dried aerial parts of *E. alata* monjauzeana were grinded into a powder using a Microfine grinder Merke IK MF 10 Basic Staufen (DE) Germany. The EamCE was obtained by means of maceration with a mixture of methanol/water (80:20, *v*/*v*), under constant stirring and left overnight in the dark. The maceration exudate was filtered, and the recovered solution was then evaporated under vacuum using a rotary evaporator at 35 °C. The process was repeated every 24 h, three times. The EamCE was dissolved in a small quantity of methanol/water (80:20, *v*/*v*) and eventually filtered through a 0.2 μm filter before its analysis.

### 2.4. RP-HPLC-ESI-QTOF-MS Analysis

MS Analyses were performed with an Agilent 1200 series rapid resolution (Agilent Technologies, Palo Alto, CA, USA) supplied by a binary pump, an auto sampler, and a diode array detector (DAD), using a quadrupole-time-of-flight mass spectrometry analyzer (QTOF, model 6540 Agilent Ultra-High-Definition Accurate-Mass Q-TOF), equipped with an electrospray ionization interface (ESI, model Agilent Dual Jet Stream interface).The flow amount was adjusted at 0.80 mL/min throughout the gradient. Then, 10 µL of the EamCE solution (20.000 mg/L) was injected. Separation was executed on a 150 × 4.6 mm, 1.8 µm, Zorbax Eclipse Plus C18 column (Agilent Technologies) at room temperature. Gradient elution was run, utilizing as eluent A: water with 0.1% formic acid and as eluent B: acetonitrile. The following multistage linear gradient was applied: 0 min, 5% B; 45 min, 100% B; 55 min, 5% B; and, finally, a conditioning cycle of 5 min, with the same conditions for the next analysis. The separated compounds were monitored in sequence first with the DAD and then with a mass spectrometry detector spectra that were acquired over a mass range from *m*/*z* 70 to 1100 operating in negative ionization mode. Internal mass correction was achieved with an unceasing infusion of Agilent TOF mixture consisting of trifluoracetic acid, ammonium salt, and hexakis (1H,1H,3H-tetrafluoropropoxy) phosphazine. All spectra were calibrated prior to phytochemical identification. The detection window was set to 100 ppm. The MS and MS/MS data were processed using the Mass Hunter Qualitative Analysis B.06.00 software (Agilent Technologies) that yielded a list of eventual elemental formulas.

### 2.5. TPC, TFC, In Vitro Antioxidant Photoprotective and Anti-Enzymatic Activities

All experiments were realized in 96-well microplates, and the absorbance measurements were carried out on a Multimode Plate Reader, EnSpire, PerkinElmer, Waltham (US) United States of America. BHA, BHT, α-Tocopherol, ascorbic acid, tannic acid, gallic acid, quercetin, galantamine, acarbose, kojic acid, thiourea, and orlistat were used as standards (positive controls) to estimate the relative extract activity. The EamCE solution was prepared at seven different concentrations, (800, 400, 200, 100, 50, 25, 12.5 µg/mL) and every assay was realized in triplicate. The results were expressed as 50% inhibition (IC_50_) and absorbance at 0.5 (A0.5) concentrations that are able to inhibit/block/chelate 50% or reduce the absorbance to 0.5 of the radical, enzyme catalytic sites or the formation of metallic complexes/cations.

#### 2.5.1. Total Phenolic Content (TPC) and Total Flavonoid Content (TFC) Assessment

##### TPC Dosage

The total phenolic content was assessed spectrophotometrically according to Folin–Ciocalteu method, modified by References [16,17], and the result was expressed as micrograms of gallic acid equivalents per milligram of extract (μg GAE/mg).

##### TFC Dosage

The total flavonoid content was assessed spectrophotometrically following the method described by Topçu et al. [18], and the result was expressed as micrograms quercetin equivalents per milligram of extract (μg QE/mg).

#### 2.5.2. Estimation of Antioxidant Activities

##### ABTS Scavenging Procedure

The ABTS^•+^ scavenging ability was performed spectrophotometrically according to the modified procedure of Re et al. [19]. First, the ABTS^•+^ was prepared as follows: 2 mM of ABTS was dissolved in H_2_O with 2.45 mM potassium persulfate (K_2_S_2_O_8_), and the mixture was conserved at ambient temperature for 16 h in obscurity. Second, 160 μL of diluted ABTS^•+^ solution (delivering an absorbance value of 0.700 ± 0.025 at 734 nm) was added to 40 μL of EamCE solution. Thereafter, the microplate was incubated for 10 min before measuring the absorbance at 734 nm. The equation below (*) was used to calculate the inhibition percentage of ABTS radical, and results were presented as IC_50_ values.

I% = ((Ac − As)/Ac) × 100 (*)(1)

I: inhibition;

Ac: control’s absorbance;

As: sample’s absorbance.

##### DPPH Scavenging Procedure

The scavenging capacity of the stable DPPH free radical was indicated by the adjusted method of Blois [20]: 160 µL of DPPH solution (1 mM) in methanol was put in reaction with 40 µL of the EamCE solution; then, absorbance was measured at 517 nm after 30 min of incubation in the dark. Results were provided as IC_50_ values, and the I% were calculated using the above formula (*).

##### Superoxide Alkaline DMSO Test

The superoxide radical was produced as described Kunchandy’s method [21]: 30 µL of NBT (nitroblue tetrazolium) (1 mg/mL) and 40 μL of sample were added to 130 μL alkaline DMSO (1.0 mL DMSO, 5 mMNaOH, 100 µL H_2_O). The absorbance of the reaction mixture was measured at 560 nm, and results were provided as IC_50_ values.

##### Reducing Power Test

To assess the reducing power effect, 10 μL of the EamCE solution were added to 40 μL of 0.2 M phosphate buffer (pH 6.6) and 50 μL of potassium ferricyanide (1%), incubated for 20 min at 50 °C. Later, 50 μL of TCA (trichloroacetic acid) (10%) and 10 μL of ferric chloride (0.1%) were added before finally measuring the mixture’s absorbance at 700 nm. The results were given as the EamCE concentration, giving an absorbance 0.5 (A0.5) [22].

##### β-Carotene/Linoleic Acid Bleaching Test

Proceeding as described by Marco [23], with minor changes: 0.5 mg of β-carotene, 1 mL of chloroform, 25 μL of linoleic acid, and 200 μL of Tween 40 were added, forming an emulsified mixture. Then, it was evaporated under vacuum, and 50 mL of hydrogen peroxide (30%) were added with vigorous shaking. The absorbance at 470 nm should give a value between (0.8–0.9). Next, 160 μL of the prepared β-carotene was added to 40 μL of EamCE solution. The 0 min (t_0_) and 120 min (t_120_) time absorbances were measured, and the results were given as IC_50_ values, according to the following equation:

I%= 1 − ((As (t_0_) − As (t_120_))/(Ac (t_0_) − Ac (t_120_))) × 100 (2)

where As is the absorbance of the tested sample at t0 (time = 0 min) and A (time = 120 min) of the reaction, and Ac is the absorbance of control (methanol) at t0 (time = 0 min) and A (time = 120 min) of the reaction.

##### Cupric Reducing Antioxidant Capacity (CUPRAC) Assay

The method described by Reference [24] was used with no modifications. In brief, 10 mM of CuCl_2_ (50 μL), 7.5 mM of neocuproine in ethanol (50 μL), and 1 M of CH_3_COONH_4_ (60 μL) with 40 μL of the EamCE solution was added simultaneously to generate the reaction, and the mixture was incubated for 1 h before measuring the absorbance at 450 nm. The result was given as A0.5 value.

##### Hydroxyl Radical Scavenging Assay

It was evaluated according to the modified method of Smirnoff and Cumbes [25]. Initially, 40 μL of the EamCE solution was mixed with 80 μL salicylic acid (3 mM), 24 μL FeSO_4_ (8 mM), and 20 μL H_2_O_2_ (20 mM). The microplate was incubated for 30 min at 37 °C, and 36 μL H_2_O was added; immediately, the absorbance was measured at 510 nm. The result was given as IC_50_ value.

##### O-Phenanthroline Assay

As proceeded by Szydlowska-Czerniaka et al. [26], the reaction mixture held 30 μL o-phenanthroline (0.5% in methanol), 50 μL FeCl_3_ (0.2%), 110 μL methanol, and 10 μL of the EamCE solution. Next, it was incubated for 20 min at 30 °C before measuring the absorbance at 510 nm. The result was given as A0.5 value.

##### Galvinoxyl Radical (GOR) Scavenging Assay

The procedure consisted of adding 160 µL of (0.1 mM) galvinoxyl in methanol to 40 µL of the EamCE solution, followed by an incubation of 120 min, and then the absorbance was read at 428 nm. The result was given as IC_50_ value [27].

##### Silver Nanoparticle-Based Method

The reduction of Ag^+^ to spherical silver nanoparticles (SNPs) was developed by Özyürek et al. [28]. Firstly, 130 µL of SNP solution (prepared by heating 50 mL of AgNO_3_ (1.0 mM) for 10 min; then, 5 mL of trisodium citrate (1%) was added drop by drop until a pale-yellow color was obtained) and 50 µL of H_2_O were added to 20 µL of the EamCE solution. The microplate was incubated for 30 min at 25 °C, and the absorbance was read at 423 nm. The result was given as A0.5 value.

#### 2.5.3. In Vitro Photoprotective Capacity

The photoprotective property of the EamCE was determined following the procedure reported by Cristina et al. [29]. It is expressed by the sun protection factor (SPF). The sample was dissolved in methanol at a concentration of 2 mg/mL (2000 ppm). Afterwards, the absorbance was recorded at seven different wavelengths with a 5 nm interval, from 290 to 320 nm.

SPF is the ratio calculated using the formula below:

SPF spectrophotometric = CF × ∑ EE (λ) × I (λ) × Abs (λ)(3)

EE: erythemal effect spectrum, I: solar intensity spectrum, Abs: absorbance of sunscreen product.

CF: correction factor (= 10).

The values of EE(λ) × I(λ) are constants determined by Sayre et al. [30] and are displayed in Table 1.

#### 2.5.4. Anti-Enzymatic Activities

##### Evaluation of Anti-Acetylcholinesterase (AChE) and Anti-Butyrylcholinesterase (BChE) Activities

Anti-AChE and Anti-BChE inhibitory assays were evaluated using the method described previously in the work of Ellman and Öztürk [31,32]. Briefly, 150 µL of 100 mM sodium phosphate buffer (pH 8.0), 10 µL of the EamCE solution, and 20 µL AChE (5.32 × 10^−3^ U) or BChE (6.85 × 10^−3^U) were mixed. At this point, the mixture was incubated for 15 min at 25 °C; additionally, 10 µL of DTNB (0.5 mM) was added, and 10 µL of acetylthiocholine iodide (0.71 mM) or 10 µL of butyrylthiocholine chloride (0.2 mM) was added to initiate the reaction. The results were given as IC_50_ values.

##### Anti-Alpha Amylase Potential

The anti-alpha amylase potential was assessed using the iodine/potassium iodide method, with few modifications [33]. To begin, the sample (25 μL) was mixed with an α-amylase solution (1U (50 μL)) and then incubated at 37 °C for 10 min. After that, the reaction was started by adding a starch solution (50 μL, 0.1%). Concurrently, a control was prepared without putting the enzyme solution. After another incubation of 20 min at 37 °C, 25 μL HCl (1 M) and 100 μL iodine-potassium iodide solution were added successively to stop and assess the reaction by measuring the absorbances at 630 nm, and result was given as IC_50_ value.

##### Tyrosinase Inhibition Ability

Tyrosinase inhibition ability was performed employing L-DOPA as substrate as detailed before [34]. First, 10 μL of the EamCE solution was mixed with 150 μL of sodium phosphate buffer (100 mM, pH 6.8) and 20 μL of tyrosinase enzyme solution (150 units/mL) and incubated for 10 min at room temperature. Then, 20 μL of L-DOPA (5 mM) was added to initiate the reaction; after that, the mixture was incubated further for 10 min at 37 °C before measuring the absorbance at 475 nm. The result was given as IC_50_ value.

##### Urease Inhibition Capability

Urease inhibitory capability was determined by measuring ammonia production using the indophenol method [35]. Briefly, the reaction mixture consisted of 25 μL of enzyme solution ((5 U/mL) (Jack bean urease)), 10 μL of the EamCE solution, and 50 μL of urea substrate solution, then it was incubated at 30 °C for 15 min. Then, 45 μL of phenol reagent (2 g of phenol (C_6_H_5_OH) in 25 mL H_2_O + 25 mg of Na_2_[Fe(CN)_5_NO],2H_2_O in 25 mL H_2_O) and 70 μL of basic reagent (0.7125 g of NaOH in 25 mL H_2_O + 1.175 mL of NaOCl in 25 mL H_2_O) were added later. After 50 min of incubation, the absorbance was measured at 630 nm, and result was given as IC_50_ value.

##### Inhibitory Pancreatic Lipase Activity

Porcine pancreatic lipase (PPL, type II) activity was assessed employing p-nitrophenyl butyrate (p-NPB) as a substrate, according to the slightly modified protocols [36,37]. First, 100 μL of enzyme solution (1 mg/mL in 50 mM Tris-HCl (pH 8.0)) was added to 50 μL of the EamCE solution. Then, the microplate was kept for 20 min at 37 °C. Finally, 50 μL of p-NPB (5 mM) was added to initiate the reaction.

##### Anti-Inflammatory Test by Heat-Induced Hemolysis Method

An erythrocyte suspension was prepared as follows: a total human blood was obtained from a safe donor and then centrifuged for 5 min at 3000 rpm in heparinized centrifuge tubes. The suspension was washed three times with an equivalent volume of NaCl 0.9%. Subsequently, it was diluted to obtain a suspension of 10% (*v*/*v*) in an isotonic buffer solution (10 mM sodium phosphate buffer, pH 7.4). The procedure consists of adding 0.05 mL of the erythrocyte suspension and 0.05 mL of the EamCE solution mixed with 2.95 mL phosphate buffer (pH 7.4). The conical tubes were incubated at 54 °C for 20 min in a shaking water bath. Once this was done, they were centrifuged at 2500 rpm for 3 min, and the absorbance of the supernatant was measured at 540 nm against a control using phosphate buffer. Diclofenac (purchased from a pharmacy) was used as a reference compound [38]. The EamCE percentage inhibition was recorded according to the equation below:

% inhibition of hemolysis = 100 − (1 × C/S)(4)

where C = absorption of the control, and S = absorption of test sample mixture.

#### 2.5.5. Cytotoxic Test

##### Cell Culture

Hep2 (Human epithelial type 2 (laryngeal carcinoma)) and Rd (rhabdomyosarcoma) ×cells were kindly provided by Pasteur Institute, Algiers (DZ), Algeria. The cells were grown and maintained in Dulbecco’s Modified Eagle Medium (DMEM) supplemented with 10% (*v*/*v*) fetal calf serum and 1% (*v*/*v*) antibiotic–antimycotic in a 37 °C, 5% CO_2_ humidified atmosphere. The cells were harvested every 3 days. After thawing, the cells were kept in normal culture conditions for 10 days before experiments.

##### Cytotoxicity Assessment

Hep2 and Rd cells were incubated with different concentrations (15.625, 31.25, 62.5, 125, 250, 500 μg/mL) of the EamCE for 48- and 72-h periods. Hep2 and Rd cell viability was assessed by the MTT assay [39], where 100 μL of MTT was added and incubated at 37 °C for 4 h. The insoluble formazan was dissolved in 100 μL of DMSO. The absorbance was measured at 490 nm. All experiments and measurements were performed in triplicate.

The cytotoxic effect was determined using the below formula:

Cytotoxic effect (% Cell inhibition) = 1 − (Ac/As) × 100(5)

Ac: absorbance of the control;

As: absorbance of the sample.

### 2.6. Statistical Analysis

Results are expressed as the mean values ± SD of three measurements; the IC_50_ and A0.50 values were calculated by linear regression analysis, and variance analyses were performed by ANOVA using XLSTAT. Significant differences between means were determined by Tukey test, and *p* values ˂0.05 were regarded as significant.

## 3. Results

**Figure 1:**
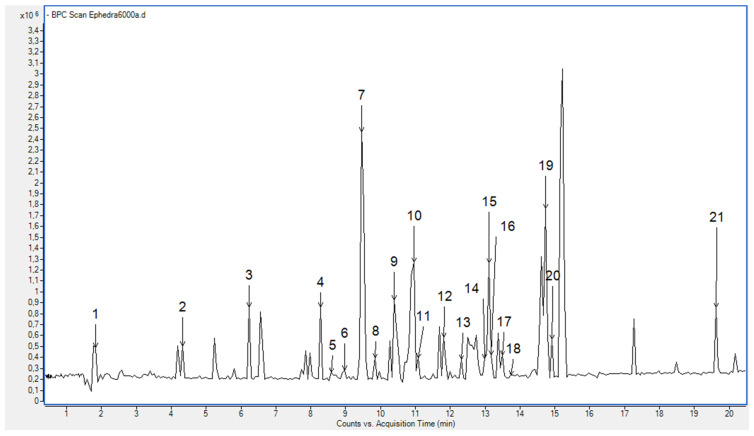
BPC of the EamCE.

The base peak chromatogram (BPC) of the EamCE in the negative ionization mode is presented in Figure 1. A list of the molecular ions ([M − H]^−^) found in the EamCE and the proposed tentative identification is given in Table 2. Twenty-one compounds were identified and characterized on the basis of their MS and MS/MS data (Figure 1).

**Figure 2:**
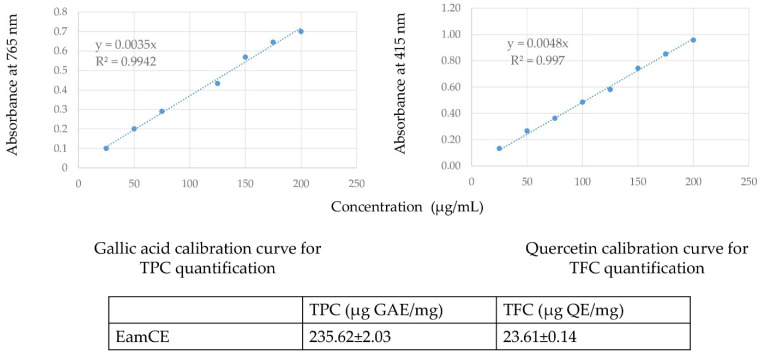
TPC and TFC results. TPC is expressed as μg gallic acid equivalents/mg of extract (μg GAE/mg). TFC is expressed as μg quercetin equivalents/mg of extract (μg QE/mg).

The quantification of both EamCE phenolic and flavonoid contents, additionally, to calibration curves of gallic acid and quercetin are shown in Figure 2.

Results of in vitro antioxidant and anti-enzymatic tests are reported as the mean values ± SD of three measurements; the IC_50_ and A0.5 values were calculated by linear regression analysis and are summarized in Table 3 and Table 4, respectively. The EamCE SPF value is demonstrated in Table 5, with reference product SPF values.

In regard to antioxidant EamCE potential, in almost all tests, the IC_50_ and A0.50 values were close to the standard ones, except for the beta-carotene bleaching test and hydroxyl scavenging assays, that showed moderate effects.

Similarly, for the anti-enzymatic activities, the EamCE IC_50_ value was less than the standard one for anti-BChE test and very near for the anti-AChE test. However, using the alpha amylase test, the reaction of the EamCE gave the best response, with a much lowerIC_50_ value than that of acarbose. In addition, the anti-urease and anti-tyrosinase IC_50_ values were also very near to those of the reference compounds. For the anti-lipase test, the EamCE has exhibited an interesting inhibiting effect of 50% of lipase available in the mixture, with only 50 µg/mL (Table 4).

The heat-induced hemolysis (in-vitro anti-inflammatory test) results are illustrated in Table 6, where the EamCE has given a favorable activity. Finally, cytotoxic activity was expressed by the calculation of the inhibition percentage of cancerous cell lines. Effectively, no sufficient cell killing potential was recorded. The results are presented in Table 7.

## 4. Discussion

The identified phenolic compounds of the EamCE were categorized into three classes: phenolic acids, phenylpropanoids, and flavonoids.

Three compounds corresponding to peaks 1, 2, and 5 (caffeic acid [40], gallic acid [13], and *O*-coumaric acid glucoside [41] (glycosylated)) are phenolic acids. Only compound 16 has been identified as a phenylpropanoid [40].

Many different flavonoids have been found, some of them belonging to the subclass of flavonols, and they were allocated to glycosylated compounds; we identified: myricetin-*O*-hexoside [42], quercetin-*O*-rhamnoside [43], hyperoside [44], quercetin-3-*O*-galactoside [45], isorhamnetin-3-*O*-glucoside [44], kaempferol rhamnoside [41], quercetin-3-*O*-glucoside [46] (corresponding to peaks 11, 12, 13, 15, 17, 19, 20, respectively). Di-glycosylated flavonols were also found in peaks 6 and 10 (quercetin 3-*O*-rhamnoside-7-*O*-glucoside [47], rutin [44]).

Besides, the flavones were di-glycosylated compounds, namely apigenin-6,8-C-dihexoside [42] (peak 7), apigenin 6-C-pentoside-8-C-hexoside [47] (peak 9), glycosylated compound that was designated as luteolin 8-C-glucoside [47] (peak 14), and to non-glycosylated compound, which is the last flavone “luteolin” [42] (peak 21).

Likewise, the analysis has shown the presence of three flavan-3-ols that are recorded in peaks 3, 4, and 8, appropriately: (epi)gallocatechin [41], catechin-*O*-hexoside (glycosylated) [42], and epi-catechin [48].

Only one structure was defined as flavanone “naringenin-*O*-hexoside”(peak 18) [42].

Numerous studies have affirmed the presence of phenolic acid and flavonoid compounds in different *Ephedra* species, notably *E. alata*. These compounds act as the main contributors to the antioxidant potential and to many other biological activities of plant extracts [12,49,50]. Flavonoids are the most common class of secondary metabolites within the genus of *Ephedra*, and over forty flavonoids have been classified as flavonols, dihydroflavonols, flavonones, flavanols, flavones, and anthocyanins; notably, flavones and their glycosides, as well as flavonols and their 3-*O*-glycosides constituents, are the most common flavonoids in *Ephedra* [51]. It has been discovered in *Ephedra* species that certain glycans (ephedran A, B, C, D, and E) occurred in the aerial parts, and diverse flavanols were identified to be components of twigs and barks [52]. Furthermore, several additional secondary metabolites originating from the molecular identification of different *Ephedra* species in previous studies included alkaloids, amino acids and derivatives, volatiles, and phenolic compounds. The alkaloids were of significant biological relevance: ephedrine, pseudoephedrine, norephedrine, norpseudoephedrine, methylephedrine, and methylpseudoephedrine. Other alkaloids have been detected in some Eurasian *Ephedra* species, such as: ephedroxane and macrocyclic spermidines called ephedradine A–D [53]; nevertheless, they were not found in our plant.

The TPC and TFC analyses have shown that the EamCE possesses a considerable amount of phenolic and flavonoid compounds, which are higher than the ones found in other studies of the same plant species [54]. The plausible antioxidant potential of a bioactive product can be estimated by distinct in vitro assays based on the inhibition ability of consistent free radicals [55]. To that end, we have proceeded by several distinct antioxidant tests with direct, indirect, or competitive procedures, reduction, chelation, or inhibition principles. We believe that the ten tested methods could ensure the antioxidant properties of the EamCE, even if it gives a medium potential for one of them. Effectively, the EamCE has provided a very powerful antioxidant activity, and the IC_50_ and A0.5 concentrations shown in Table 3 were relatively low and approximately the same order of magnitude than the standard ones (except for the β-carotene bleaching and hydroxyl scavenging assays). Presumably, the quantity of the molecule responsible of the effect is lower, if not considering that the measured activity was a result of the synergic effect of many combined molecules, even though the EamCE abilities to act as an antioxidant product remained very substantial and neighboring those of reference compounds. The EamCE demonstrated a strong antioxidant capacity with lower DPPH IC_50_ (µg/mL) and reducing power A0.5 (µg/mL) values comparatively to other extracts from *E. alata*, *E. alata Campylopoda Fragilis* (ECF), *Procera fisch.* et may (EPfm), and *sarcocarpa* (Es) species of different previous studies. Indeed, DPPH IC_50_ from *E. alata* extract was >1000, 83.07 ± 0.2 [49], 330 ± 0.004, 454 ± 0.008, 180 ± 0.002, 176± 0.002 [12], 450 ± 7, 540 ± 3.455 ± 6 [42]. DPPH IC_50_ (µg/mL) from ECF extracts was 125 ± 4.4, 150 ± 5.1, 300 ± 4.4 [56]. DPPH IC_50_ (µg/mL) from EPfm extract was 300 ± 4.4, 150 ± 5.1, 125 ± 4.4 [56,57]; and DPPH IC_50_ (µg/mL) from Es extract was 5300 ± 0.027 [58]. Moreover, reducing power A0.5 (µg/mL) from *E. alata* plant extracts was 108 ± 1 [50], 377 ± 4 [42]. Referring to earlier antioxidant studies of *E. alata* species or other species, none of them have used ten different antioxidant methods to demonstrate how powerful and strongly bioactive is the extract of interest. The exhibited prominent antioxidant activity of the EamCE enables us to consider it as a very promising product that could be involved in the resolution of many physiological disorders related to oxidant antioxidant imbalances and oxidative stress that induce the development of others serious pathologies.

For the current time, we hardly found reports about enzymatic inhibition properties for the family of *Ephedra* and its species; we aimed across this work to reveal the ability of this plant to block the catalytic site of distinct enzymes. Therefore, it would be valuable and relevant for application in the context of pathologic dysfunction of the enzymes in question.

Today, the neurodegenerative disorder Alzheimer’s disease is more frequent in older people worldwide, and the conception of efficient treatments requires a perfect awareness of the physiological mechanisms involved [59]. An AChE hyperactivity was mentioned in the brain of Alzheimer affected subjects, particularly in the senile plaque-rich fraction [60]. AChE preponderates in the healthy brain, contrary to BChE, that slightly influences the regulation of brain acetylcholine amounts. However, BChE activity progressively increases in patients with AD. Both enzymes constitute legitimate therapeutic targets for enhancing the cholinergic deficit causing the decline in cognitive, behavioral, and global functioning features of AD [61]. The EamCE has showed an impressive inhibitory potential (Table 4) of both AChE and BChE enzymes, particularly for the BChE blocking effect, that has given an IC_50_ (µg/mL) value (28.91 ± 1.08) better than that of galantamine (34.75 ± 1.99). This leads us to fully consider that the chemical constituents are sufficiently able to act as AChE and BChE antagonists; by the same token, researchers have already proven that selected phenolic acids and flavonoids exhibited an important inhibitory activity towards AChE and BChE, and these compounds are in common with the chemical constitution of the EamCE, for instance: caffeic acid, gallic acid, apigenin, quercetin, luteolin, and kaempferol [62,63].

Diabetes is a metabolic dysfunction defined by chronic hyperglycaemia. It exists several and various medical approaches for the cure of type 2 diabetes. The inhibition of alpha-amylase activity is only one way to downregulate postprandial blood glucose levels [64,65]. The inhibitors of this enzyme can be used in the treatment of obesity and diabetes. In diabetic patients, it catalyzes the hydrolysis of α-(1,4)-d-glycosidic linkages of starch and other glucose polymers. The inhibition of α-amylase prevents starch breaking and results in lower levels of blood glucose [66]. A previous study has demonstrated the role of flavonoids in the inhibition of alpha-amylase activity, and it has also qualified the common flavonol myricetin (one of the EamCE components) as a strong flavonoid inhibitor of α-amylase and considered the possibility to deliver it in concentrated form (capsules with food intake) to reduce postprandial hyperglycaemia (by inhibiting starch digestion) [67]. The action mechanism proposed for the inhibitory ability of flavonoids is associated with the number of hydroxyl groups on the B ring of the flavonoid skeleton, the formation of hydrogen bonds between the hydroxyl groups of the polyphenol ligands, and the catalytic residues of the binding site of the enzyme [68]. The important inhibitory ability is noticed in flavonols and flavones groups [68,69,70]. Our finding is in agreement with the preceding ones; the EamCE has presented an impressive antidiabetic activity that was more efficient than the one of acarbose, and we have noted a very large difference comparing their IC_50_ (µg/mL): 22.66 ± 0.17, 3650.93 ± 10.70, respectively.

Tyrosinase is a crucial enzyme of melanin metabolism implicated in identifying the color of mammalian skin and hair. The accumulation of an excessive level of epidermal pigmentation, due to tyrosinase hyperactivity, causes different dermatological disorders, such as melasma, age spots, and sites of actinic damage, besides the unfavorable enzymatic browning of plant-derived foods that induce the decrease in nutritional quality and economic loss of food products [71]. Previously, enzyme kinetics and molecular docking techniques were used to examine the inhibitory capacity and the structural mechanism of flavonoids on tyrosinase; according to the obtained favorable results, authors suggested that flavonoids containing an additional hydroxyl group at the C-3′ position, and especially a 3′,4′-dihydroxyl substitution on ring B, increase tyrosinase inhibitory effects and can be considered as potential candidates for the design of tyrosinase inhibitor drug [72]. Since the EamCE contained high concentrations of flavonoids, it has exhibited a remarkable tyrosinase blocking activity.

Inhibition of the metalloenzyme urease has considerable pharmacological utilizations in the matter of antiulcer and anti-gastric cancer drugs. Urease is involved in many serious infections caused by *Helicobacter pylori* in the gastric tract, as well as by *Proteus* and related species in the urinary tract [73]. In a similar manner to other enzymatic inhibitory activities, the EamCE has demonstrated a blocking property of the urease catalytic site.

Obesity is a multifactorial pathology defined by an overweight to height ratio, depending on an intensified fat deposition, such as adipose tissue, which is related to more excessive calorie consumption than energy expenditure [74]. The imbalance between calorie consumption and metabolic expenditure is a central factor in several cases of obesity, and reduction in the intake of energy dense fats may be useful to reduce weight; therefore, the suppression of energy intake by inhibiting the action of pancreatic lipase that splits triglycerides into absorbable glycerol and fatty acids could be a useful strategy [75]. Phytochemicals identified from traditional medicinal plants are biologically active and can act as antiobesity agents. Various plants have been screened for their anti-lipase activity due to the abundance of inhibitors from different chemical classes: saponins, polyphenolics, terpenes and triterpenes [74,76]. Orlistat is an antiobesity agent, which selectively and potently inhibits the absorption and the hydrolysis of fat that results in 30% decrease in fat absorption [77]. In our work, it has given (orlistat) an outstanding effect (IC_50_: 0.061 ± 0.001 µg/mL); however, the EamCE was not that efficient, but it has performed a remarkable and distinguishing anti-lipase potential comparatively to plenty of other plant extracts that have exhibited low effectiveness in many potent anti-obesity agents reports [78,79,80,81,82,83,84].

Long exposure to UV radiation increases the risk of skin diseases, such as cancer and photoallergic reactions. UV-B (280–320 nm) radiation is mainly responsible for inducing skin problems. Natural substances have been recently considered as potential sunscreen resources due to their absorption in the UV region and their antioxidant activity. Clearly, a good correlation was found between SPF and phenolic contents of plant extracts [85], due to that fact the EamCE has given an important index of photo screening estimated by an SPF of 29.20 ± 0.92, which is considered moderate, but not negligible, comparatively with commercial and cosmetic sun screen SPF values (Table 5).

Recent findings have demonstrated that quercetin-rich methanol extract of *Ephedra ciliata* has an anti-inflammatory activity, which promoted the healing of wounds in two different models, and, at cytokine reduced amount, the downregulation of TNF-α was suggested as the inducer factor of the anti-inflammatory and wound healing activity [86]. Critical compounds of *Ephedra*, including quercetin, luteolin, kempferol, naringenin, and beta-sitosterol, were identified in treating asthma by inhibiting the expression of many anti-inflammatory targets, SELE, IL-2, and CXCL10, at mRNA and protein levels; these substances are involved in the biological processes of immune response, inflammatory response, cell-cell signaling, and response to lipopolysaccharide [87]. In our anti-inflammatory approach, the EamCE has provided a convincing ability to inhibit hemolysis induced by heating. The percentage of inhibition was very high, at 76.76 ± 0.15%, and, at a very low concentration of the EamCE (65.5 µg/mL), it has shown the same effect as the anti-inflammatory drug used as a reference (Diclofenac), notably at the same concentration. Clearly, the effect has not been reduced, even in low concentration (71.03 ± 1.38% at 8.1875 µg/mL), as shown in Table 6, and we proclaim that this eminent effect is due to the presence of the affirmed anti-inflammatory compounds discussed in the above research works.

A recent in-depth analysis reported on the screening of extracts from 57 plants for checking the relationship between free radical scavenging and cytotoxicity. The results revealed that the extracts of plants exhibited an EC_50_ of free radical scavenging ≤10 μg/mL showed a degree of enhancement in increased cytotoxicity [88].

Some compounds isolated from *Ephedra* extracts of different species, such as herbacetin, ephedrine alkaloids, and oligomeric proanthocyanidins, have exerted a putative antiproliferative potential against different cancer cell lines [51]. The criteria of cytotoxic activity for the crude extracts, as handled by the American National Cancer Institute, is an IC_50_ < 30 µg in the preliminary assay [89]. Consequently, we consider that the EamCE is not an interesting anticancer agent, contrary to other crude extracts from *E. alata* (possessing a powerful anticancer activity) [10,49,54]. The EamCE has given about 20% as an inhibition percentage at the extreme concentration of (500 µg/mL), and the same effect remained and did not change after 72 h (Table 7). This is maybe due to the non-existence of specific *Ephedra* alkaloids that engender the pharmacological and toxicological effects and induce metabolic pathways occurring in particular *Ephedra* species [53].

## 5. Conclusions

The prominent antioxidant, anti-enzymatic (neuroprotective, anti-diabetic, dermoprotective, anti-infectious, anti-obesity), and anti-inflammatory in vitro effects exerted by the EamCE parallel its rich chemical arsenal. The chemical profile is individualized by the identified flavonoids and phenolic acids (phenolic acids, phenylpropanoids, flavonoids, flavones, flavanols, flavanones). The latter are the potential candidates of the current proven biological activities, as well as its tolerable non-cytotoxicity.

The present outcomes highlight *E. alata* monjauzeana as a putative promising natural source to design food additives as a spice, preservative, essential oil, etc. Otherwise, it could be used as a dietary supplement, acting as therapeutic agent for a wide array of human illnesses or cosmetic products (such as sunscreen), with no harmful side effects.

## Figures and Tables

**Table 1:** Correlation between the erythemogenic effect (EE) and the radiation intensity at each wavelength (I) [29].

Wavelength λ (nm)	EE (λ) × I (λ) (Normalized)
290	0.0150
295	0.0817
300	0.2874
305	0.3278
310	0.1864
315	0.0837
320	0.0180
Total	1

**Table 2:** RP–UHPLC–ESI–QTOF–MSn data of flavonoids and phenolic acids identified in the aerial parts of *E. alata* monjauzeana.

Peak	Compounds	Rt (min)	Molecular Formula	*m*/*z* Experimental	*m*/*z* Calculated	Ionization Mode	Error	Major Fragments (Intensity%) *m*/*z*
1	Caffeicacid	1.90	C_6_H_12_O_6_	179.0558	179.0561	N	1.82	135 (6.7)
2	Gallicacid	4.37	C_7_H_6_O_5_	169.0143	169.0142	N	−0.39	125 (85), 79 (100)
3	(epi)gallocatechin	6.238	C_15_H_14_O_7_	305.0668	305.0667	N	−0.29	125 (3.69)
4	Catechin-O-hexoside	8.222	C_21_H_24_O_11_	451.1243	451.1246	N	0.51	289 (10.41)
5	o-Coumaric acid glucoside	8.594	C_15_H_18_O_8_	325.0928	325.0929	N	3.32	290 (15.02), 145 (8.31), 93 (57.82)
6	Quercetin 3-O-rhamnoside-7-O-glucoside	8.718	C_27_H_30_O_1_	609.1465	609.1461	N	−0.53	462 (1.82)
7	Apigenin-6,8-C-dihexoside	9.462	C_27_H_30_O_15_	593.1523	593.1512	N	−1.64	473 (53), 383 (26), 353 (44)
8	Epi-catechin	9.895	C_15_H_14_O_6_	289.0718	289.0718	N	0.23	245 (3.8)
9	Apigenin 6-C-pentoside-8-C-hexoside	10.39	C_26_H_28_O_14_	563.1413	563.1413	N	−0.99	473 (1.1)
10	Rutin	10.94	C_27_H_30_O_16_	609.1468	609.1468	N	−1.05	300 (26)
11	myricetin-O-hexoside	11.507	C_21_H_20_O_13_	479.0835	479.0831	N	−0.55	317 (6.45)
12	Quercetin-O-rhamnoside	11.817	C_21_H_20_O_11_	447.0938	447.0933	N	−1.14	300 (3.61), 173 (11.85), 111 (10.77)
13	Hyperoside	12.313	C_21_H_20_O_12_	463.0885	463.0882	N	−0.36	300 (4.31), 271 (1.09)
14	Luteolin 8-C-glucoside	12.994	C_21_H_20_O_11_	447.0935	447.0938	N	−0.38	429 (1.82)
15	Quercetin-3-O-galactoside	13.118	C_21_H_20_O_12_	463.0888	463.0882	N	−1.25	301 (4.54)
16	Verbascoside	13.181	C_27_H_28_O_17_	623.1256	623.1254	N	−0.26	461 (4.74)
17	Isorhamnetin-3-O-glucoside	13.490	C_22_H_22_O_12_	477.1043	477.1038	N	−0.6	300 (5.18)
18	Naringenin*-O-*hexoside	13.676	C_21_H_22_O_10_	433.1134	433.114	N	0.91	271 (15.2)
19	Kaempferolrhamnoside	14.792	C_21_H_20_O_10_	431.0986	431.0984	N	−0.5	285 (4.74)
20	Quercetin-3-O-glucoside	14.91	C_21_H_20_O_12_	463.0883	463.0882	N	−0.02	300 (39)
21	Luteolin	19.68	C_15_H_10_O_6_	285.0406	285.0405	N	−0.57	133 (2)

**Table 3:** Antioxidant potentials with IC_50_ and A0.5 values.

Products	DPPH (IC_50_)	ABTS (IC_50_)	CUPRAC (A0.5)	Reducing Power (A0.5)	Beta Carotene (IC_50_)	DMSO Alcalin (IC_50_)	SNP (IC_50_)	Phenonthroline (A0.5)	GOR (IC_50_)	Hydroxyl Radical (IC_50)_)
EamCE	32.49 ± 0.49 ^a^	11.77 ± 0.81 ^a^	25.71 ± 1.66 ^a^	38.57 ± 1.44 ^a^	380.96 ± 0.93 ^a^	15.31 ± 0.91	30.97 ± 0.87 ^a^	17.11 ± 0.30 ^a^	31.38 ± 0.56 ^a^	163.32 ± 1.39 ^a^
BHT *	12.99 ± 0.41 ^b^	1.29 ± 0.30 ^b^	8.97 ± 3.94 ^b^	NT	1.05 ± 0.03 ^b^	NT	NT	2.24 ± 0.17 ^b^	5.38 ± 0.06 ^b^	NT
BHA *	6.14 ± 0.41 ^c^	1.81 ± 0.10 ^c^	5.35 ± 0.71 ^c^	NT	0.91 ± 0.01 ^c^	NT	NT	0.93 ± 0.07 ^c^	3.32 ± 0.18 ^c^	NT
α-Tocopherol *	13.02 ± 5.17 ^d^	NT	NT	34.93 ± 2.38 ^b^	NT	˂3.125	NT	NT	NT	NT
Ascorbic Acid *	NT	NT	8.31 ± 0.15 ^d^	6.77 ± 1.15 ^c^	NT	NT	7.14 ± 0.05 ^b^	3.08 ± 0.02 ^d^	5.02 ± 0.01 ^d^	32.33 ± 1.17 ^b^
Tannic Acid *	NT	NT	NT	5.39 ± 0.91 ^d^	NT	<3.125	NT	NT	NT	NT
Trolox *	5.12 ± 0.21 ^e^	3.21 ± 0.06 ^d^	8.69 ± 0.14 ^e^	5.25 ± 0.20 ^e^	NT	NT	34.17 ± 1.23 ^c^	5.21 ± 0.27 ^e^	4.31 ± 0.05 ^e^	NT

* Standard compounds. NT: not tested. IC_50_ and A0.50 values are defined as the concentration of 50% inhibition percentages and the concentration at 0.50 absorbance, respectively. IC_50_ and A0.50 were calculated by linear regression analysis and expressed as mean ± SD (*n* = 3). The values with different superscripts (^a^, ^b^, ^c^, ^d^, ^e^) in the same columns are significantly different (*p* < 0.05).

**Table 4:** Anti-enzymatic results (IC_50_ µg/mL).

Products	Anti-AChE	Anti-BChE	Anti-Alpha Amylase	Anti-Tyrosinase	Anti-Urease	Anti-Lipase
EamCE	22.46 ± 0.91 ^a^	28.91 ± 1.08 ^a^	22.66 ± 0.17 ^a^	38.04 ± 0.98 ^a^	23.55 ± 1.04 ^a^	54.93 ± 0.17 ^a^
Galantamine *	6.27 ± 1.15 ^b^	34.75 ± 1.99 ^b^	Na	Na	Na	Na
Acarbose *	Na	Na	3650.93 ± 10.70 ^b^	Na	Na	Na
Kojic acid *	Na	Na	Na	25.23 ± 0.78 ^b^	Na	Na
Thiourea *	Na	Na	Na	Na	11.57 ± 0.68 ^b^	Na
Orlistat *	Na	Na	Na	Na	Na	0.061 ± 0.001 ^b^

* Standard compounds. IC_50_ is defined as the concentration of 50% inhibition percentage. Na: no activity. IC_50_ was calculated by linear regression analysis and expressed as mean ± SD (*n* = 3). The values with both superscripts (^a^, ^b^) in the same columns are significantly different (*p* < 0.05).

**Table 5:** The EamCE photoprotective activity.

Wavelength EE × I(nm)	EamCE	Nivea *	Vichy *
290 0.015	0.53 ± 0.01	0.77 ± 0.00	0.66 ± 0.00
295 0.0817	2.75 ± 0.04	4.48 ± 0.00	3.64 ± 0.01
300 0.2874	8.94 ± 0.24	14.39 ± 0.00	12.67 ± 0.10
305 0.3278	9.37 ± 0.35	16.17 ± 0.23	14.47 ± 0.11
310 0.1864	5.01 ± 0.17	9.35 ± 0.28	8.25 ± 0.08
315 0.0837	2.13 ± 0.08	4.04 ± 0.00	3.69 ± 0.01
320 0.018	0.43 ± 0.01	0.87 ± 0.02	0.80 ± 0.00
SPF	29.20 ± 0.92	50.10 ± 0.53	44.22 ± 0.34

* Reference compounds.

**Table 6:** Heat induced hemolysis (percentage inhibition of hemolysis).

Concentration (µg/mL)	Diclofenac% Inhibition	EamCE% Inhibition
65.5	73.87 ± 1.86 ^a^	76.76 ± 0.15 ^a^
32.75	72.05 ± 0.64 ^a^	76.11 ± 2.08 ^b^
16.375	72.03 ± 0.49 ^a^	74.92 ± 1.21 ^b^
8.1875	70.23 ± 0.99 ^a^	71.03 ± 1.38 ^b^

The inhibition % of hemolysis is expressed as mean ± SD (*n* = 3). The values with different superscripts (^a^, ^b^) in the same line are significantly different (*p* < 0.05), and the values with the same superscripts (^a^, ^a^) are not significantly different (*p* > 0.05).

**Table 7:** The EamCE percentage inhibition of HEP2 and RD cell lines.

	HEP2 (% Inhibition)		RD (% Inhibition)	
Concentration (µg/mL)	48 h	72 h	48 h	72 h
500	25.25 ± 0.06 ^a^	28.56 ± 0.05 ^a^	7.20 ± 0.04 ^a^	12.56 ± 0.45 ^a^
250	22.02 ± 0 ^b^	22.87 ± 0.05 ^b^	6.4 ± 0.21 ^b^	9.67 ± 0.23 ^b^
125	19.78 ± 0.04 ^c^	19.32 ± 0.04 ^c^	3.02 ± 0.06 ^c^	5.11 ± 0.94 ^c^
62.5	6.74 ± 0.05 ^d^	10.8 ± 0.08 ^d^	1.22 ± 0.05 ^d^	3.42 ± 0.12 ^d^
31.25	2.81 ± 0 ^e^	10.23 ± 0.05 ^e^	0 ^e^	1.11 ± 0.02 ^e^
15.625	0 ^f^	9.52 ± 0.07 ^f^	0 ^f^	0 ^f^

IC_50_ are expressed as mean ± SD (*n* = 3). The values with different superscripts (^a^, ^b^, ^c^, ^d^, ^e^, ^f^) in the same columns are significantly different (*p* < 0.05).

## Data Availability

Not applicable.
